# Structured literature review of patient-reported outcome (PRO) instruments in adult tonsillectomy or tonsillotomy

**DOI:** 10.1186/s12955-019-1192-z

**Published:** 2019-07-15

**Authors:** Alicia Seethaler, Claudia Rudack, Christoph Spiekermann

**Affiliations:** 1 Department of Otorhinolaryngology, Head and Neck Surgery, University Hospital Münster, Münster, Germany; 2 Institute of Immunology, University Hospital Münster, Röntgenstr. 21, 48149 Münster, Germany

**Keywords:** Tonsillectomy, Tonsillotomy, Outcome, Patient-reported outcome measure, Review

## Abstract

**Background:**

Instruments that measure the patient-reported outcome and quality of life are essential to assess the treatment success of any medical intervention. This review represents valid and reliable outcome assessment instruments for tonsillectomy (TE) and tonsillotomy (TO) in adult patients as TE/TO still belong to one of the most common performed surgical procedures.

**Methods:**

A systematic review of the literature in the MEDLINE, PubMed, Web of Science and Cochrane Library was conducted. Studies describing reliable and valid patient-reported outcome measures (PROM) in adults with regard to the perioperative as well as postoperative follow-up after TE/TO were examined. Thus, studies without PROMs or PROMs only relating to children as well as studies in non-English/non-German language or without any detailed information were excluded.

**Results:**

Four thousand four hundred forty studies were identified. Thirteen reliable and valid patient–reported outcome assessment instruments presenting the perioperative and postoperative outcome were analysed. Four generic questionnaires are included that are used to measure the outcome after TE/TO in adults. Four disease-specific questionnaires relating to obstructive sleep apnea (OSA) and sleep disordered breathing (SDB) as well as two TE/TO specific questionnaires are validated for adults. With regard to the perioperative outcome including parameters like pain, nausea, vomiting, satisfaction three assessment instruments are analysed.

**Conclusion:**

This review describes the currently available, reliable and valid generic and disease-specific instruments assessing the perioperative as well as postoperative outcome to evaluate the treatment success after TE/TO in adult patients. Therefore, this study improves the selection of the appropriate patient–reported outcome assessment instrument to assess the quality of life in adults undergoing TE/TO.

## Background

Tonsillectomy (TE) and tonsillotomy (TO) (TE/TO) belong to the most common operations [1, 2]. The most frequent indications are infections like recurrent tonsillitis and peritonsillar abscess as well as sleep-disordered breathing (SDB) including the obstructive sleep apnea (OSA) [3].

Tonsillectomy does not only have a huge impact on the health-related quality of life (HRQOL) of children but also affects the outcome in adults. However, the number of studies that measure the outcome and benefit in adult patients is still lower than in paediatric ones. There are significant differences between adult and paediatric attitudes, especially in the assessment of the HRQOL, which have to be considered in the evaluation of the outcome [4].

Children for example, are primarily influenced by their social environment consisting of their family, school and peer-group. Health-related restrictions that influence these child contexts like playing with friends result in a worse assessment of HRQOL. There are differences in the rating of the HRQOL of the children between parents and children themselves because their perception of HRQOL itself as well as language comprehension distinguishes [4, 5]. Furthermore, the own perception of HRQOL changes with the developmental level for children of different ages as well [6]. In addition, the consequences of the adverse effects caused by the symptoms of recurrent tonsillitis, sore throat episodes and SDB vary greatly in dependence on the patient’s age. In contrast, for adult patients, absence from work and lack of concentration has an influence on productivity and consequently on the socio-economic status. Job insecurity has a great impact on the quality of life and the health status [7]. In 2016 the German Federal Statistical Office DESTATIS published the number of inpatient treatments due to chronic diseases of palatine and pharyngeal tonsils. In total 98506 inpatient treatments were recorded in 2015 in Germany. Thereof, 49812 patients are younger than 15 years while 48694 are above 15 years. In addition, it has to be considered that chronic diseases of pharyngeal tonsils/adenoids are included as well and therefore, the percentage of tonsillitis is probably higher among patients older than 15 years [8]. These data impressively indicate the high impact of recurrent tonsillitis in the adult patients which has been kept unattended in the current literature so far.

HRQOL questionnaires ought to measure HRQOL by its multidimensional aspects while integrating objective as well as subjective domains that consist of physical-, social-, emotional-wellbeing and development and activity [9]. These domains are important to evaluate the treatment’s efficiency as they involve the patient-reported outcome as well [10]. Furthermore, the subjective outcome parameters are only observable and discernible with a self-report of the patient. Nowadays, the evidence of an improvement of the HRQOL is a basic prerequisite in assessing the benefit on any medical intervention [11–13].

The aim of this review article is to present the reliable and valid outcome assessment instruments for adults with regard to the perioperative as well as postoperative follow-up after TE and TO.

## Materials and methods

Based on the preferred reporting items for systematic reviews and meta-analyses (PRISMA) guidelines a structured research in the MEDLINE, PubMed, Web of Science and Cochrane Library database was conducted using the following combinations of search terms: “Tonsillectomy” AND “Quality of life” OR “Tonsillotomy” AND “Quality of Life” as well as “Tonsillectomy” AND “Outcome” OR “Tonsillotomy” AND “Outcome” [14]. Detailed information concerning the search strategy are illustrated in Table 1. Literature from the inception of the database to September 15, 2017 was included in the present study and literature research has been updated on January 22, 2019.

**Table 1:** Search strategy to identify studies using instruments to assess the patient-reported outcome and HRQOL in Adults undergoing tonsillectomy or tonsillotomy

Database	Strategy
Pubmed/MEDLINE (*n* = 2112)	Search terms: “Tonsillectomy” AND “Quality of life” OR “Tonsillotomy” AND “Quality of Life” as well as “Tonsillectomy” AND “Outcome” OR “Tonsillotomy” AND “Outcome” in all fields. Literature from inception of the database to January 22, 2019.
Cochrane Library (*n* = 575)	Search terms: “Tonsillectomy AND Quality of life”, “Tonsillotomy AND Quality of Life” as well as “Tonsillectomy AND Outcome”, “Tonsillotomy AND Outcome” in all fields. Literature from inception of the database to January 22, 2019.
Web of Science (*n* = 1753)	Search terms: “Tonsillectomy AND Quality of life”, “Tonsillotomy AND Quality of Life” as well as “Tonsillectomy AND Outcome”, “Tonsillotomy AND Outcome” in all fields. Literature from inception of the database to January 22, 2019.

### Inclusion criteria

Studies utilizing patient-reported outcome measures (PROM) for patients older than 18 years were included. Validity and reliability of questionnaires which were used to assess the outcome of patients undergoing TE/TO because of recurrent episodes of acute tonsillitis, hyperplastic tonsil, sleep-disordered breathing and peritonsillar abscess were examined. Therefore, the reliability was considered to be “good”, if one type of reliability has been tested with satisfactory results (e.g. internal consistency or test-retest reliability), and if both types have been tested with at least good results the reliability was rated as “very good”. Validity was considered “good” if one or two types of validity have been tested with satisfactory results (e.g. content and construct validity) and if more than two types of validity have been tested with at least good results, validity was rated “very good” [11]. (Table 2) Perioperative as well as postoperative follow up studies were analysed.

**Table 2:** Patient-reported outcome measures in TE/TO validated for adults

		PROM	Indication	Frequency used in Studies	Recall Period used in Studies	Validity	Reliability	Responsiveness	Development	Reference
Postoperative Outcome	*Generic Questionnaires*	GBI	Generic outcome in otorhinolaryngological surgery	12	6 m - 4 y	+	+	+	Robinson et al.	[15–17]
		WHOQOL-BREF	Assessment of QoL	1	6–8 m	++	++	+	WHOQOL Group	[18–20]
		SF-36	Assessment of general health	7	6 m - 1 y	++	++	+	RAND Corporation	[21–23]
		15D-HRQOL	Assessment of health-related QoL	2	6 m - 2 y	++	+	+	Sintonen	[24]
	*OSA/SDB-Specific Questionnaires*	ESS	SDB	25	6 w - 3 m	+	+	+	Johns et al. (1)	[25–28]
		SAQLI	SDB	1	1 y	++	++	++	Flemons et al.	[29, 30]
		Berlin-Questionnaire	OSA	1	2–6 m	+	+	0	Netzer et al.	[31, 32]
		FOSQ	OSA	4	3–6 m	++	++	+	Weaver et al.	[33–35]
	*TE/TO-Specific Questionnaires*	TAHSI	Tonsil and adenoid disease	1	6 m - 1 y	++	++	++	Steward et al.	[36]
		TOI-14	Chronic tonsillitis	2	1 m - 1 y	++	++	++	Skevas et al.	[12]
Perioperative Outcome		VAS/NRS	Outcome after surgery	59	1 d - 2 y	+	+	+		[37, 38]
		PONV		1	1 h - 2 d	+	++	++	Wengritzky et al.	[39]
		QUIPS		1	1 d	+	+	0	Meissner et al.	[40]

Reliability: + (one type of reliability has been tested with satisfactory results e.g. internal consistency or test-retest reliability), ++ (both types have been tested with at least good results)Validity: + (one or two types of validity have been tested with satisfactory results e.g. content and construct validity), ++ (more than two types of validity have been tested with at least good results)Responsiveness: + (responsiveness has been tested), ++ (responsiveness has been tested with good to very good results), 0 (responsiveness has not been assessed) Frequency Used in Studies: Number of studies identified in our research that utilize these questionnaire for adult patients

*Abbreviations*: *TE* Tonsillectomy, *TO* Tonsillotomy, *OSA* Obstructive Sleep Apnea, *SDB* Sleep Disordered Breathing, *QoL* Quality of Life, *GBI* Glasgow Benefit Inventory, *WHOQOL-BREF* World Health Organization Quality of Life abbreviated version, *SF-36* Short-Form 36 Survey, *15D-HRQO* 15 Dimensions Health-Related Quality of Life, *ESS* Epworth Sleepiness Scale, *SAQLI* Calgery Sleep Apnea Quality of Life Index, *FOSQ* Functional Outcomes of Sleep Questionnaire, *TAHSI* Tonsil and Adenoid Health Status Instrument, *TOI-14* Tonsillectomy Outcome Inventory 14, *VAS* Visual Analogue Scale, *NRS* Numeric Rating Scale, *PONV* Postoperative Nausea and Vomiting intensity scale, *QUIPS* Quality Improvement in Postoperative Pain Management, *h* hour, *d* days, *w* weeks, *m* months, *y* years

### Exclusion criteria

**Fig. 1:**
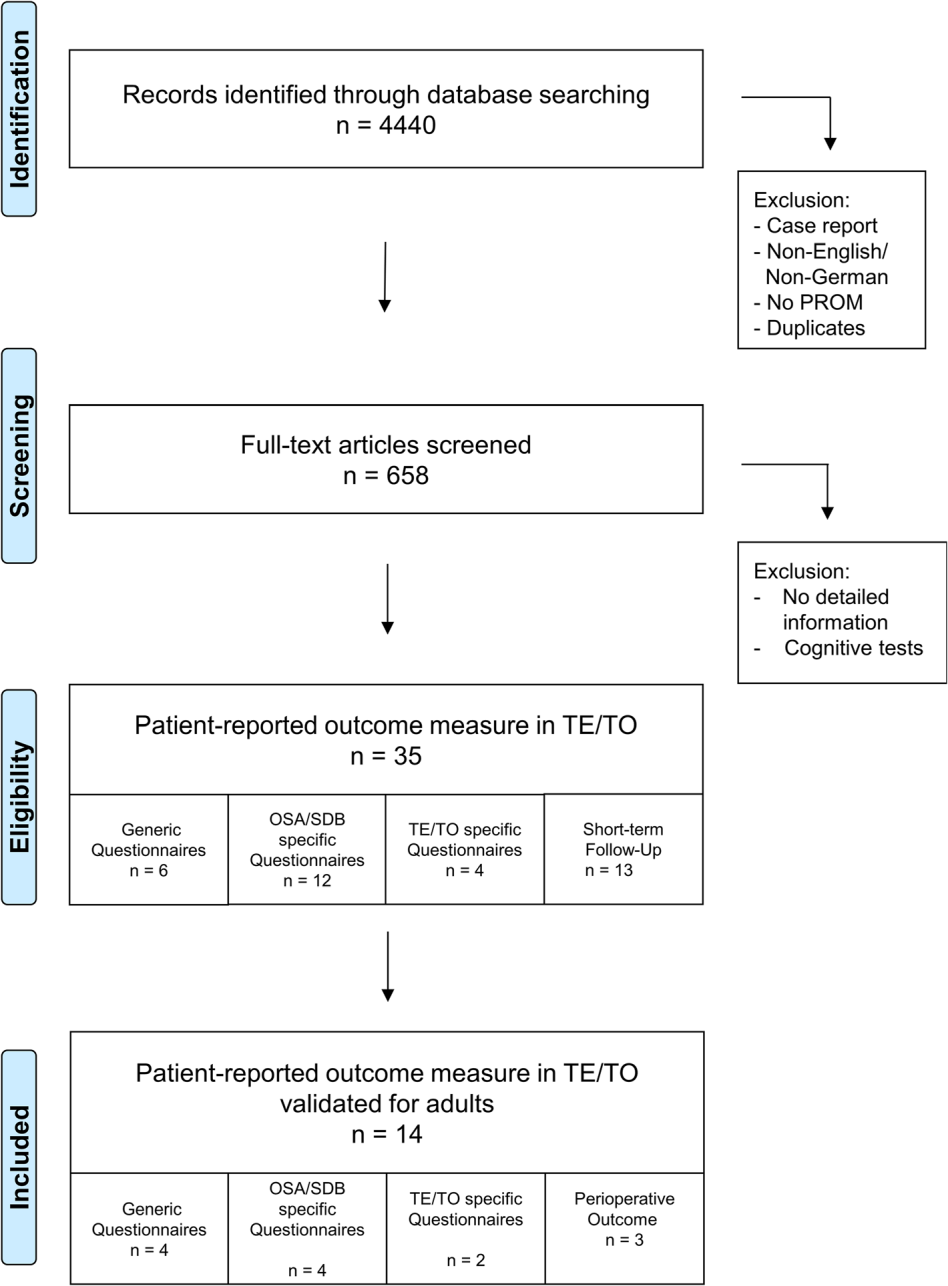
PRISMA flow diagram illustrating research strategy. Abbreviations: TE = Tonsillectomy; TO = Tonsillotomy; OSA = Obstructive Sleep Apnea; SDB = Sleep Disordered Breathing

Studies describing non-patient-related outcome parameters were excluded as well as studies in non-English/non-German language, case reports and duplicates. Systematic reviews, literature reviews and meta-analyses were excluded to avoid duplicates. Studies with questionnaires only validated for paediatric patients younger than 18 years and questionnaires regarding cognitive behaviour were rejected. Furthermore, non-validated questionnaires and studies without available detailed information about validity and reliability were excluded. Figure 1 illustrates the study selection process of the database research.

## Results

Using the different combinations of search terms, 4440 studies could be identified. After excluding duplicates and screening the titles and abstracts 658 studies remained for further analysis. Additionally, studies without detailed information and studies that measure the cognitive behaviour were rejected afterwards. Of the remaining studies all questionnaires were analysed and divided into different categories:

1. Generic questionnaires
2. OSA/SDB specific questionnaires
3. TE/TO specific questionnaires
4. Perioperative follow up

Finally, the questionnaires were analysed with regard to their validation in adult patients. In total, four generic questionnaires, four OSA/SDB and two TE/TO specific questionnaires as well as four questionnaires measuring the perioperative follow up met the inclusion criteria (Table 2).

### Generic questionnaires

In this review four validated generic questionnaires which were used to assess the outcome of TE/TO in adult patients could be identified. The Glasgow Benefit Inventory (GBI), the World Health Organization Quality of Life (WHOQOL-BREF), the Short-Form 36 Survey Version 2 (SF-36) and the 15 Dimensions Health-Related Quality of Life (15D-HRQOL) were included. The number of questions ranged from 15 to 36 while the number of domains varies from three to 15.

The GBI is a measure of the generic patient-reported outcome that was developed for otorhinolaryngological interventions by Robinson et al. in 1996. It is only conducted once postoperatively and self-completed by the patients (above 18 years) or during an interview in order to identify the changes in the health status due to different interventions [15]. These interventions are not just regarded to surgical procedures but also include medical interventions. The GBI consists of 18 items that are divided into three subscales: the general subscale consists of twelve questions about general and psychosocial health; the social scale reports the need of social support using three more questions; and the physical health subscale includes the three remaining questions about consultations of physicians or medication requirements [16, 41]. Questions are answered using a five-point Likert scale with a range from one to five, while a score of one is indicating the worst change of health status and a score of five the best change. Afterwards the responses of the 18 questions are summed up and divided by 18 to obtain an average score. Subtracting three of this score and then multiplying by 50 leads to the finally score with a range from − 100 (indicating the poorest outcome) through zero (no change) to + 100 (best outcome) [15, 16]. The GBI is a reliable and validated instrument measuring changes in the health status and HRQOL after surgical interventions. The whole questionnaire as well as the subscales themselves are reliable, valid and sensitive to represent changes in general patient-reported outcome [15].

The WHOQOL-BREF is the short version of the WHOQOL-100 which is a cross-culturally validated assessment of well-being and both are developed by the WHOQOL Group [18]. The WHOQOL-100 contains 100 items in six domains that represent 24 facets [42]. Therefore, the WHOQOL-BREF uses one item of each already existing facet and additionally two items of the overall quality of life and general health facet. In total, the WHOQOL-BREF represents a 26-item instrument with four different domains: domain one represents the physical health while domain two examines the psychological aspects. Domain three presents social relations and domain four reflects the environment [43]. The different questions are answered using four different five-point Likert interval scales, which are originally used by the WHOQOL-100. These scales reflect intensity, capacity, frequency and evaluation as they illustrate “how much”, “how completely”, “how often”, “how good” or “how satisfied” the patient felt during the last 2 weeks. Afterwards the scores are converted into a scale from zero to 100 [18, 44]. The questionnaire is self-completed by the respondent (above 18 years) but can also be answered during an interview.

The WHOQOL-BREF is a reliable and validated instrument to assess the HRQOL while representing good discriminant validity, content validity, internal consistency and test-retest reliability as a short version of the WHOQOL-100 [18, 42].

The Short-Form 36 Health Survey Version 2 (SF-36) is a generic questionnaire measuring the well-being and health of the respondent. It consists of eight health domains: Physical Functioning, Role-Physical, Bodily Pain, General Health, Vitality, Social Functioning, Role-Emotional and Mental Health which all contribute to the physical component summary scores (PCS) and mental component summary (MCS) scores. It has different applications including measuring changes in the health status and the treatment effectiveness, predicting medical expenses and comparing disease burden in different populations. In total, 36 questions are asked and the questionnaire is available in 170 translations for adult respondents above 18 years [45, 46]. The questionnaire is answered by the patients themselves or during an interview by using different response choices including different five-point Likert scales and yes/no answers [46]. Afterwards, the given answers are transformed using a scoring key with possible scores from zero to 100 which presents the achieved percentage. Higher scores indicate a better health status [47]. In addition, the SF-36 is one of the most used patient-reported outcome instruments overall [48]. For adults, the SF-36 represents a reliable and valid questionnaire measuring physical and mental health [21–23, 46].

The 15 dimensions health-related quality of life (15D-HRQoL) instrument represents the health status of a person or a group as a profile. It is used as a self-administration but the questions can also be answered during an interview. In total, the 15D-HRQoL consists of 15 dimension including mobility, vision, hearing, breathing, sleeping, eating, speech, excretion, usual activities, mental function, discomfort and symptoms, depression, distress, vitality and sexual activity. Answers are given using different five-ordinal levels on each dimension scoring one to five [24, 49]. With the help of a special valuation system the questionnaire is scored from zero to one while zero means being dead and one indicates having no problems on any dimension (“full HRQOL”) [50]. Actually, the questionnaire is available in 31 different languages and is developed for persons above 16 years. The questionnaire is able to identify the actual health status of a person or a group as a profile and as a single index score. Therefore, it represents a reliable and valid method as it has high content validity as well as construct validity. In addition, the 15D-HRQoL has a high sensitivity as the discriminatory power as well as the responsiveness to change have been proven [24, 50, 51].

### OSA/SDB specific questionnaires

Four disease specific questionnaires which are indicated for OSA or SDB in adults were included in this review. Therefore, the Epworth Sleepiness Scale (ESS), the Berlin-Questionnaire, the Sleep Apnea Quality of Life Index (SAQLI) and the Functional Outcomes of Sleep Questionnaire (FOSQ) were analysed. The number of questions ranged from eight to 35 while the number of domains varies from three to five.

The ESS was developed in 1990 by Johns et al. in order to assess the daytime sleepiness of the respondents. The questionnaire consists of eight questions that are answered by the patients themselves. The answers are given using a four-point scale with a range from zero to three (0 = would never doze; 1 = slight chance of dozing; 2 = moderate chance of dozing; 3 = high chance of dozing). Therefore, the total score varies from zero to 24 with higher scores indicating a higher average sleep propensity (ASP) of a person’s daily live. The ESS is an instrument measuring the daytime sleepiness in eight different situations while disregarding subjective feelings and the duration of drowsiness [52]. The ESS is a reliable instrument in measuring persistent daytime sleepiness in adult because it has a high internal consistency as Cronbach’s alpha scores r = 0.88 [25]. Furthermore, the external criterion validity of the ESS has been evaluated with the help of a functional MRI study [53] and the Multiple Sleep Latency Test (MSLT) [54]. Additionally, the responsiveness of the ESS questionnaire to treatment effects has been tested for obstructive sleep apnea [26, 52]. However, the significant correlations between the ESS scores and the MSLT scores and the severity of sleep apnea were rebutted in more recent studies [27] but the studies analysed in this review generally used the ESS in addition to polysomnography findings and that’s why the ESS is not used as a diagnostic tool by itself.

The Calgery Sleep Apnea Quality of Life Index (SAQLI) is a disease-specific questionnaire developed by Flemons and Reimer in order to measure the quality of life in adults with sleep apnea or any sleep disorders. It consists of 35 questions containing four domains: daily functioning with eleven items, social interactions including thirteen questions, emotional functioning with eleven items and symptoms with regarding five questions. Treatment-related symptoms including five more questions can be used as an additional domain for active therapy and therapeutic interventions like surgery. This questionnaire is answered during an interview with a trained interviewer. Therefore, three different response options using different coloured seven-point Likert scales scoring one to seven were used while the respondents are able to add other symptoms if necessary [29]. Scoring one indicates maximal impairment while seven states no impairment. The questionnaire is available in three different languages. It is related with a high internal consistency (Cronbach’s alpha = 0.88 to 0.92) as well as a high construct validity and responsiveness [29, 30]. Thus, the SAQLI is a reliable and valid disease-specific questionnaire measuring quality of life in adult patients with sleep apnea in clinical trials.

The Berlin-Questionnaire is a disease-specific questionnaire identifying patients with obstructive sleep apnea. It consists of ten questions organized in three different categories that are answered by the respondents themselves. Category one examines the frequency and presence of sleep and snoring behaviour. Category two evaluates the fatigue or daytime sleepiness while the last category identifies possible hypertension and obesity [31, 55]. The questionnaire is scored with the help of a scoring algorithm and is considered positive if two or more categories are scored positive. Category one and two are positive if two or more answers are positive while category three is positive if one answer is positive or the BMI is greater than 30. A positive questionnaire indicates a high risk of having sleep apnea while a negative one (one or no categories are positive) suggests low risk [31, 56]. The Berlin-Questionnaire is a reliable validated instrument assessing the risk for sleep apnea in adult patients. It has a high internal consistency with Cronbach’s alpha varying from 0.86 to 0.92 [31]. In total, the Berlin-Questionnaire has good sensitivity for identifying OSA in sleep clinic population [57, 58] but it depends on the definition of OSA as the sensitivity varies with different hypopnea definitions [32].

The Functional Outcomes of Sleep Questionnaire (FOSQ) is a disease-specific questionnaire for adults representing the impact of disorders of excessive sleepiness (DOES) in different daily activities and how these disorders are improved by treatment. The questionnaire consists of 30 questions organized into five subscales: activity level, vigilance, intimacy and sexual relationships, general productivity and social outcome. Answers are given self-administered using a four-point rating scale from zero to four (no difficulty, a little difficulty, moderate difficulty, extreme difficulty). If respondents have no difficulties in the stated activity they ought to skip the next questions while respondents who have difficulties are asked to score how often they have these difficulties on four-point scale (once in a while, some of the time, most of the time, all the time). Additionally, patients are asked how frequently they perform special activities on a six-point scale from zero to five (never did it – three or more times a week) [33]. A shorter version of the FOSQ, the FOSQ-10 has been developed as well [59]. The FOSQ is a reliable instrument as Cronbach’s alpha value varies from 0,87 to 0,95 for the whole test and the subscales yield scores higher than 0.7. Furthermore, it is a valid questionnaire as it has high discriminant and construct validity and its concurrent validity has been successfully proven using the SF36 and SIP questionnaires [33, 34].

The short form FOSQ-10 is a reliable and valid quality of life instrument to determine functional health status in adults as well [59].

### TE/TO specific questionnaires

Apart from the already contemplated questionnaires, there are another two disease-specific questionnaires that are specific to tonsillectomy (TE) and tonsillotomy (TO) and validated for adult patients. Hence, the Tonsil and Adenoid Health Status Instrument (TAHSI) and the Tonsillectomy Outcome Inventory 14 (TOI-14) are analysed in this review.

The TAHSI is a disease-specific QoL questionnaire that measures the outcome in adult patients with tonsil and adenoid disease. Although there exists a version for adults, the English version of the TAHSI developed by Stewart et al. in 2001 has been only validated for children aged two to 16 years [60, 61]. Therefore, the German version (G-TAHSI) is examined here as it is validated for adult patients. Originally, the THASI consists of six subscales but in the German version three subscales were added though the final number of 18 questions sustained. Thus, the nine subscales include recurrent throat infections, halitosis, chronic throat infection, swallowing problems, lymphadenopathy, health care utilization, severe throat infections, work performance and nocturnal breathing with two questions per subscale. The questions are answered during a telephone interview using the original five-point Likert scale scoring zero (no problem) to four (very severe problems). Afterwards the achieved points are summed up with a final range from zero to 72 while lower scores indicate a low burden of disease, an improved HRQOL and a better outcome [36, 61]. The G-THASI is a reliable and validated outcome assessment instrument for adult patients with chronic or recurrent tonsillitis. Internal consistency was proven by a Cronbach’s alpha of 0.92 and a reliability coefficient of 0.89 could be determined. Guyatt’s Responsiveness Index is 5.1 indicating a good responsiveness. Furthermore, the TAHSI has a specificity of 90% and sensitivity of 80% [36].

The TOI-14 is a disease-specific questionnaire for adults with chronic tonsillitis that measures the HRQOL in the long-term period. It consists of 14 questions which are organized into four subscales: throat discomfort (questions one to four); general health (questions five and six); resources (questions seven to ten) and social psychological restriction (questions 11 to 14). Questions are answered using a six-point Likert scale with a score from zero (no problem) to five (couldn’t be worse). Afterwards the achieved points of each subscale are summed up, divided by the number of questions and multiplied by 100. Thus, there is a score for each subscale as well as a total score each with a range from zero to 100 with higher scores indicating a higher burden of disease. The TOI-14 is a reliable and valid disease-specific questionnaire that describes the HRQOL in adult with chronic tonsillitis. Its reliability was measured by calculating Cronbach’s alpha for the total score (0.86) and the subscales (range from 0.68 to 0.9) indicating a moderate to good internal consistency. Moreover, the test-retest-reliability was discovered whereas the subscores “general health” and “resources” represent moderate, the subscore “throat discomfort” a good and the subscore “social psychological restriction” and the total score a very high test-retest-reliability. The content validity, the discriminant validity, the convergent validity and the sensitivity of the questionnaire were detected. The discriminant validity seems to be excellent as the control group had much less complaints than patients with chronic tonsillitis. The convergent validity shows a good conformity with regard to the total score and the subscore “general health” while the remaining subscores only have a moderate one. The sensitivity to detect clinical improvement after surgeries was tested by using the standardized response mean (SRM) and demonstrated major effects postoperatively [12].

### Perioperative follow up

This review represents four instruments in order to identify the perioperative outcome parameters like dysphagia, pain, nausea and emesis. Therefore, these questionnaires were used to reflect the chronological sequence and development after medical interventions like surgery. Here, the visual analogue scale (VAS) as well as the Numeric Rating Scale (NRS), the Postoperative Nausea and Vomiting (PONV) intensity scale and the Quality Improvement in Postoperative Pain Management (QUIPS) are described.

One of the most common used instruments is the VAS and the NRS. Both are self-administered by the respondent with higher scores displaying greater pain intensity. Additionally, both are available horizontally and vertically as they are a one-dimensional measures of pain. Using the VAS, the patient is asked to point his pain intensity with a pencil on a line from zero to 100. Measuring the distance with a ruler identifies the patient’s subjective score while zero indicates no pain and 100 demonstrates pain as bad as it could be [37, 62, 63]. The VAS is a reliable and valid method in representing the pain intensity in adults. Nevertheless, the reliability is higher among literate than illiterate patients [37, 64, 65]. There is a good correlation between the scores from horizontal and vertical scales [66]. Another version of the VAS is the NRS. It usually consists of eleven items with a range from zero to ten (0 = no pain, 10 = worst pain). Thus, respondents are asked to select the number that represents their pain intensity best during the past 24 h. In contrast to the VAS, the NRS can be answered verbally during a phone interview and therefore no appearance in person is necessary [37, 67]. Furthermore, the NRS is a reliable and valid instrument as well [37, 62, 63, 65, 68]. Both, VAS and NRS are quick and easy instruments in representing the pain intensity in adults because the administrative burden is low. Besides, there are hardly any complications in translating the scales into different languages [37].

The PONV intensity scale is another instrument identifying the perioperative outcome and possible complications after surgery. The relevance and importance of nausea and vomiting is well known, as it is one of the most common complication after anaesthesia and its risk factors should not be disregarded [39, 69–71]. The PONV intensity scale consists of four questions with different response options and therefore, different scores. The first question has three response options (no = 0 points; once or twice = 3 points; three or more times = 50 points), the second question has four options (no = 0 points; sometimes = 1 point; often or most of the time = 2 points; all of the time = 25 points) and the third one has two response options (varying (“comes and goes”) = 1 point; constant (“is nearly or almost always present”) = 2 points). The last questions asked about the duration of nausea in hours and therefore, no particular score is available. A total score higher than 50 is defined as clinically important. This questionnaire is usually used 6 h after surgery and can be repeated after 24 and 72 h. The PONV intensity scale is a reliable and valid measure to detect existing nausea and vomiting after surgery because it has a high correlation with the necessary amount of antiemetic drugs and the nausea VAS score. In addition, the reliability of the PONV intensity scale is high with a score higher than 0.91 up to 0.99. The responsiveness has been proven with an excellent discriminatory ability and a large effect size of 0.82 [39].

The QUIPS is a questionnaire that represents the outcome after surgery with regard to postoperative pain, patient’s satisfaction and medication. It consists of 16 questions with different response options. Therefore, four questions about pain are answered using a numeric rating scale with a score from zero (no pain) to ten (worst pain), one question about the patient’s satisfaction is answered with a numeric rating scale from zero (very dissatisfied) to ten (very satisfied), one question about the patient’s incorporation in decision-making is answered with another numeric rating scale from zero (not at all) to ten (totally included) and the remaining eleven questions about pain, satisfaction, nausea/vomiting and well-being are answered with “yes” or “no”. The questionnaire is answered during a personal interview or by the patients themselves [72]. Apart from this the QUIPS questionnaire serves as a benchmark because the results of the different hospitals that participate on the QUIPS project were sent to a “benchmark server”. Thus, the results of the different hospitals can be compared in order to guarantee an external, subject-specific benchmarking. Due to this project, the best clinical practice can be identified to ensure a quality improvement in postoperative pain management. Nevertheless, all data except from the own results are anonymised and an anonymous peer comparison and feedback is possible [40, 73, 74]. The QUIPS questionnaire performs well in the domains of reliability and validity. Cronbach’s alpha scores 0.84 for the numeric rating scales whereas the dichotomous items score an average Kuder-Richardson-20-Coefficient of 0.52. In order to prove the validity of QUIPS the pain intensity and functional impairment from two different surgeries were compared. The two surgeries differ significantly [40, 73].

## Discussion

Nowadays, it is not sufficient and adequate to measure the success of a medical intervention and surgery without any patient-related outcome or subjective point of view. Every medical treatment is intended to improve HRQOL and (if the improvement cannot be achieved) it must at least not result in impairment. Therefore, the application of assessment instruments that measure the subjective perception is essential and even indispensable because many outcome parameters can only be assessed through self-report [75, 76]. Furthermore, the subjective and self-reported information are material to the treatment success because the patient himself as individual is ought to deal with the personal consequences of the medical intervention. In contrast to clinical standard parameters patient-reported outcomes provide an insight into personal effects and consequences of a therapy for an individual patient. Hence, individuals with the same state of health, diagnosis and diseases have different attitudes, feelings and perceptions as their own ability for coping with the present restrictions and handicap differs. Thus, the perceived influence of the disease on the patients’ satisfaction with life varies greatly. The importance to measure the well-being and HRQOL of a patient is increasing in medical intervention with the primary goal to improve the well-being itself and not to increase life expectancy. Therefore, patients with chronic diseases that are not life-threatening, such as recurrent tonsillitis, are concerned about their ability for living a life without the including restrictions whereas patients in end-stage of a disease have totally different sorrows and expectations of the treatment [77]. Thus, the questionnaires that are meant to represent the HRQOL and outcome of the patient after a medical treatment have to fulfil and cope with the possible individual expectations of the therapies and treatment success. Therefore, an objective as well as subjective evaluation of the outcome after surgery is necessary to guarantee a comprehensive measurement [78].

Regardless of the different purpose and aims of the PROMs it is indispensable to only use assessment instruments that performed well in the domains of reliability and validity. With regard to the reliability it is important to guarantee test-retest-reliability as well as internal consistency reliability in order to represent the stability of the measurement and minimizes the risk of confounding factors [75]. In general, validity consists of content, construct and criterion validity and represents how theory as well as empirical evidence are able to contribute adequate and appropriate interpretations and actions [79]. Content validity is the ability to measure the concept of interest and to forecast the future-outcome while construct validity includes convergent and divergent validity and measures if constructs are related to each other [80]. Criterion validity describes the correlation of the instrument with other validated measures, ideally in comparison with a “gold standard” [81]. Only reliable and valid PROMs are capable to ensure an accurate acquisition of data and to assess the outcome of the patient’s correctly.

This review represents the currently available PROMs for tonsillectomy that are validated for adult patients including the generic and disease-specific postoperative as well as the perioperative outcome assessment instruments. With the help of these data the selection of outcome assessment instruments for further studies is simplified because the suitable instrument can be selected depending on the particular requirements.

The generic-health questionnaires are validated instruments measuring the general quality of life and outcome after surgeries and medical interventions. Therefore, they are able to detect a range of domains including physical and emotional health and learning abilities without relating to a specific disease [4]. The different generic questionnaires are able to measure a change due to a surgical or medical intervention and provide an insight into the HRQOL of the respondents [15, 18, 21, 24, 46].

Nevertheless, generic-health questionnaires are not able to focus on specific symptoms or domains because they only measure the patient’s general well-being without relating to a small domain that is important for the clinician [82].

However, the presented non-disease-specific questionnaires are validated in general but not for the application in TE/TO, in particular. Therefore, disease-specific questionnaires that are able to focus on these specific symptoms and the associated restriction and impairment of HRQOL are designed and validated for adult patients. On the one hand OSA and SDB specific questionnaires are represented which relate to the symptoms associated with these diseases. Thus, they focus on the effects of TE/TO on OSA and SDB symptoms like sleep disturbance, physical symptoms, emotional symptoms and caregiver concerns [26, 29–31, 33, 52, 59]. On the other hand, TE/TO disease-specific questionnaires that are validated for adult patients are included. The TAHSI focuses on the outcome of patients with tonsil and adenoid disease while the TOI-14 is the worldwide only outcome assessment instrument that measures the HRQOL of patients with chronic tonsillitis [12, 36].

Apart from this, outcome assessment instruments that measure the perioperative outcome including pain, nausea, vomiting, dysphagia and patient’s satisfaction are presented. Although the burden of these side effects may not have a huge impact on the long-term outcome, it evidentially affects healing and satisfaction of the patients because it can cause significant morbidity, dehydration, bleeding and rarely life-threatening airway compromise [70, 83, 84]. Therefore, the risk factors associated with these short-term effects should not be underestimated.

In this review only English or German questionnaires are utilized in order to prevent inhomogeneity due to translations or cultures. Different nuances of language and sociocultural context in the translation of questionnaires may result in different answers and the HRQOL itself as a subjective, multidimensional experience is defined different in other cultures [85, 86].

## Conclusion

This review represents thirteen patient-reported outcome assessment instruments in English or German language that are validated for adult patients and are applicable to assess the postoperative as well as perioperative outcome after TE/TO. Thus, generic questionnaires, disease-specific questionnaire with regard to OSA/SDB or TE/TO as well as instruments measuring the perioperative follow up are included. Due to different issues and requirements in clinical practice this review will simplify the selection of the appropriate patient-reported outcome instrument.

## Data Availability

Data sharing not applicable to this article as no datasets were generated or analysed during the current study.

## Abbreviations

15D-HRQOL: 15 Dimensions Health-Related Quality of Life
ASP: Average sleep propensity
DOES: Disorders of expressive sleepiness
ESS: Epworth Sleepiness Scale
FOSQ: Functional Outcomes of Sleep Questionnaire
GBI: Glasgow Benefit Inventory
HRQOL: Health-Related Quality of Life
MSLT: Multiple Sleep Latency Test
NRS: Numeric Rating Scale
OSA: Obstructive sleep apnea
PONV: Postoperative Nausea and Vomiting
PRISMA: Preferred reporting items for systematic reviews and meta-analyses
PROM: Patient-reported outcome measurement
QUIPS: Quality Improvement in Postoperative Pain Management
SAQLI: Sleep Apnea Quality of Life Index
SDB: Sleep disordered breathing
SF-36: Short-Form 36 Survey
TAHSI: Tonsil and Adenoid Health Status Instrument
TE: Tonsillectomy
TO: Tonsillotomy
TOI-14: Tonsillectomy Outcome Inventory 14
VAS: Visual analogue scale
WHOQOL: World Health Organization Quality of Life

## References

[1] Senska G, Atay H, Putter C, Dost P (2015). Long-term results from tonsillectomy in adults. Dtsch Arztebl Int.

[2] Pynnonen M, Brinkmeier JV, Thorne MC, Chong LY, Burton MJ (2017). Coblation versus other surgical techniques for tonsillectomy. Cochrane Database Syst Rev.

[3] Erickson BK, Larson DR, St Sauver JL, Meverden RA, Orvidas LJ (2009). Changes in incidence and indications of tonsillectomy and adenotonsillectomy, 1970-2005. Otolaryngol Head Neck Surg.

[4] Matza LS, Swensen AR, Flood EM, Secnik K, Leidy NK (2004). Assessment of health-related quality of life in children: a review of conceptual, methodological, and regulatory issues. Value Health.

[5] Harding L (2001). Children's quality of life assessments: a review of generic and health related quality of life measures completed by children and adolescents. Clin Psychol Psychother.

[6] Eiser C (1997). Children's quality of life measures. Arch Dis Child.

[7] Wagenaar AF, Kompier MA, Houtman IL, van den Bossche S, Smulders P, Taris TW (2012). Can labour contract differences in health and work-related attitudes be explained by quality of working life and job insecurity?. Int Arch Occup Environ Health.

[8] Statistisches Bundesamt. Gesundheit Diagnosedaten der Patienten und Patientinnen in Krankenhäusern (einschl. Sterbe- und Stundenfälle). 2016. https://www.destatis.de/DE/Themen/Gesellschaft-Umwelt/Gesundheit/Krankenhaeuser/Publikationen/Downloads-Krankenhaeuser/diagnosedaten-krankenhaus-2120621167004.pdf?__blob=publicationFile&v=4. Accessed 29 Jan 2019.

[9] Whited JD (2015). Quality of life: a research gap in teledermatology. Int J Dermatol.

[10] World Health Organization (1997). Division of Mental Health and Prevention of Substance Abuse. WHOQOL : measuring quality of life.

[11] Kao SS, Peters MDJ, Dharmawardana N, Stew B, Ooi EH (2017). Scoping review of pediatric tonsillectomy quality of life assessment instruments. Laryngoscope.

[12] Skevas T, Klingmann C, Plinkert PK, Baumann I (2012). Development and validation of the tonsillectomy outcome inventory 14. HNO.

[13] Koller M, Lorenz W (2003). Survival of the quality of life concept. Br J Surg.

[14] Moher D, Liberati A, Tetzlaff J, Altman DG (2010). Preferred reporting items for systematic reviews and meta-analyses: the PRISMA statement. Int J Surg.

[15] Robinson K, Gatehouse S, Browning GG (1996). Measuring patient benefit from otorhinolaryngological surgery and therapy. Ann Otol Rhinol Laryngol.

[16] Hendry J, Chin A, Swan IR, Akeroyd MA, Browning GG (2016). The Glasgow benefit inventory: a systematic review of the use and value of an otorhinolaryngological generic patient-recorded outcome measure. Clin Otolaryngol.

[17] Dornhoffer JL, Smith J, Richter G, Boeckmann J (2008). Impact on quality of life after mastoid obliteration. Laryngoscope.

[18] Skevington SM, Lotfy M, O'Connell KA, WHOQOL Group (2004). The World Health Organization’s WHOQOL-BREF quality of life assessment: psychometric properties and results of the international field trial. A report from the WHOQOL group. Qual Life Res.

[19] Pibernik-Okanovic M (2001). Psychometric properties of the World Health Organisation quality of life questionnaire (WHOQOL-100) in diabetic patients in Croatia. Diabetes Res Clin Pract.

[20] Hand C (2016). Measuring health-related quality of life in adults with chronic conditions in primary care settings: critical review of concepts and 3 tools. Can Fam Physician.

[21] Stewart AL, Hays RD, Ware JE (1988). The MOS short-form general health survey. Reliability and validity in a patient population. Med Care.

[22] Stansfeld SA, Roberts R, Foot SP (1997). Assessing the validity of the SF-36 general health survey. Qual Life Res.

[23] Garratt AM, Ruta DA, Abdalla MI, Russell IT (1994). SF 36 health survey questionnaire: II. Responsiveness to changes in health status in four common clinical conditions. Qual Health Care.

[24] Sintonen H (2001). The 15D instrument of health-related quality of life: properties and applications. Ann Med.

[25] Johns MW (1992). Reliability and factor analysis of the Epworth sleepiness scale. Sleep.

[26] Hardinge FM, Pitson DJ, Stradling JR (1995). Use of the Epworth sleepiness scale to demonstrate response to treatment with nasal continuous positive airways pressure in patients with obstructive sleep apnoea. Respir Med.

[27] Chervin RD, Aldrich MS (1999). The Epworth sleepiness scale may not reflect objective measures of sleepiness or sleep apnea. Neurology.

[28] Puretic H, Plavec D, Pavlisa G, Zuljevic E, Samarzija M, Jakopovic M (2014). The Epworth sleepiness scale 23 years after: is daytime sleepiness still a valid screening tool for sleep apnea. Eur Respir J.

[29] Flemons WW, Reimer MA (1998). Development of a disease-specific health-related quality of life questionnaire for sleep apnea. Am J Respir Crit Care Med.

[30] Flemons WW, Reimer MA (2002). Measurement properties of the Calgary sleep apnea quality of life index. Am J Respir Crit Care Med.

[31] Netzer NC, Stoohs RA, Netzer CM, Clark K, Strohl KP (1999). Using the Berlin questionnaire to identify patients at risk for the sleep apnea syndrome. Ann Intern Med.

[32] Senaratna CV, Perret JL, Matheson MC, Lodge CJ, Lowe AJ, Cassim R, Russell MA, Burgess JA, Hamilton GS, Dharmage SC (2017). Validity of the Berlin questionnaire in detecting obstructive sleep apnea: a systematic review and meta-analysis. Sleep Med Rev.

[33] Weaver TE, Laizner AM, Evans LK, Maislin G, Chugh DK, Lyon K, Smith PL, Schwartz AR, Redline S, Pack AI, Dinges DF (1997). An instrument to measure functional status outcomes for disorders of excessive sleepiness. Sleep.

[34] Büttner A, Feier C, Galetke W, Ruhle K (2008). A questionnaire to capture the functional effects of daytime drowsiness on quality of life in case of obstructive sleep apnea syndrome. Functional outcomes of sleep questionnaire (FOSQ). Pneumologie.

[35] Billings ME, Rosen CL, Auckley D, Benca R, Foldvary-Schaefer N, Iber C, Zee PC, Redline S, Kapur VK (2014). Psychometric performance and responsiveness of the functional outcomes of sleep questionnaire and sleep apnea quality of life instrument in a randomized trial: the HomePAP study. Sleep.

[36] Steinbichler T, Bender B, Blassnigg E, Riechelmann H (2014). Evaluation of a German version of the tonsil and adenoid health status instrument. J Otolaryngol Head Neck Surg.

[37] Hawker GA, Mian S, Kendzerska T, French M (2011). Measures of adult pain: visual analog scale for pain (VAS pain), numeric rating scale for pain (NRS pain), McGill pain questionnaire (MPQ), short-form McGill pain questionnaire (SF-MPQ), chronic pain grade scale (CPGS), short Form-36 bodily pain scale (SF-36 BPS), and measure of intermittent and constant osteoarthritis pain (ICOAP). Arthritis Care Res.

[38] Kersten P, White PJ, Tennant A (2014). Is the pain visual analogue scale linear and responsive to change? an exploration using Rasch analysis. PLoS One.

[39] Wengritzky R, Mettho T, Myles PS, Burke J, Kakos A (2010). Development and validation of a postoperative nausea and vomiting intensity scale. Br J Anaesth.

[40] Meissner W, Mescha S, Rothaug J, Zwacka S, Goettermann A, Ulrich K, Schleppers A. Qualitätsverbesserung der postoperativen Schmerztherapie Ergebnisse des QUIPS-Projekts. Dtsch Ärztebl. 2008;105:865–70. 10.3238/arztebl.2008.0865 PMC2689629 19561807

[41] Kyrodimos E, Aidonis I, Skalimis A, Sismanis A (2011). Use of Glasgow benefit inventory (GBI) in Meniere's disease managed with intratympanic dexamethasone perfusion: quality of life assessment. Auris Nasus Larynx.

[42] Group W (1994). Development of the WHOQOL: rationale and current status. Int J Ment Health.

[43] The WHOQOL Group (1998). Anonymous Development of the World Health Organization WHOQOL-BREF quality of life assessment. Psychol Med.

[44] Szabo S, Orley J, Saxena S (1997). An Approach to Response Scale Development for Cross-Cultural Questionnaires.

[45] Optum Inc. SF-36v2 Health Survey. http://campaign.optum.com/optum-outcomes/what-we-do/health-surveys/sf-36v2-health-survey.html. Accessed 29 Jan 2019.

[46] Ware JE, Sherbourne CD (1992). The MOS 36-item short-form health survey (SF-36). I. Conceptual framework and item selection. Med Care.

[47] RAND Corporation. 36-Item Short Form Survey (SF-36) Scoring Instructions. https://www.rand.org/health/surveys_tools/mos/36-item-short-form/scoring.html. Accessed 29 Jan 2019.

[48] Scoggins JF, Patrick DL (2009). The use of patient-reported outcomes instruments in registered clinical trials: evidence from ClinicalTrials.gov. Contemp Clin Trials.

[49] Sintonen H. The 15D instrument - the health-related quality of life instrument. http://www.15d-instrument.net/15d/. Accessed 29 Jan 2019.

[50] Sintonen H (1995). The 15-D measure of health related quality of life.

[51] Sintonen H (1994). The 15D-measure of health-related quality of life.

[52] Johns MW. About the ESS. http://epworthsleepinessscale.com/about-the-ess/. Accessed 29 Jan 2019.

[53] Killgore WD, Vanuk JR, Knight SA, Markowski SM, Pisner D, Shane B, Fridman A, Alkozei A (2015). Daytime sleepiness is associated with altered resting thalamocortical connectivity. Neuroreport.

[54] Chervin RD, Aldrich MS, Pickett R, Guilleminault C (1997). Comparison of the results of the Epworth sleepiness scale and the multiple sleep latency test. J Psychosom Res.

[55] Khaledi-Paveh B, Khazaie H, Nasouri M, Ghadami MR, Tahmasian M (2016). Evaluation of Berlin questionnaire validity for sleep apnea risk in sleep clinic populations. Basic Clin Neurosci.

[56] Sert Kuniyoshi FH, Zellmer MR, Calvin AD, Lopez-Jimenez F, Albuquerque FN, van der Walt C, Trombetta IC, Caples SM, Shamsuzzaman AS, Bukartyk J, Konecny T, Gami AS, Kara T, Somers VK (2011). Diagnostic accuracy of the Berlin questionnaire in detecting sleep-disordered breathing in patients with a recent myocardial infarction. Chest.

[57] Chung F, Yegneswaran B, Liao P, Chung SA, Vairavanathan S, Islam S, Khajehdehi A, Shapiro CM (2008). Validation of the Berlin questionnaire and American Society of Anesthesiologists checklist as screening tools for obstructive sleep apnea in surgical patients. Anesthesiology.

[58] Kang K, Park KS, Kim JE, Kim SW, Kim YT, Kim JS, Lee HW (2013). Usefulness of the Berlin questionnaire to identify patients at high risk for obstructive sleep apnea: a population-based door-to-door study. Sleep Breath.

[59] Chasens ER, Ratcliffe SJ, Weaver TE (2009). Development of the FOSQ-10: a short version of the functional outcomes of sleep questionnaire. Sleep.

[60] Witsell DL, Orvidas LJ, Stewart MG, Hannley MT, Weaver EM, Yueh B, Smith TL, Goldstein NA (2008). Quality of life after tonsillectomy in adults with recurrent or chronic tonsillitis. Otolaryngol Head Neck Surg.

[61] Stewart MG, Friedman EM, Sulek M, deJong A, Hulka GF, Bautista MH, Anderson SE (2001). Validation of an outcomes instrument for tonsil and adenoid disease. Arch Otolaryngol Head Neck Surg.

[62] Huskisson EC (1974). Measurement of pain. Lancet.

[63] Downie WW, Leatham PA, Rhind VM, Wright V, Branco JA, Anderson JA (1978). Studies with pain rating scales. Ann Rheum Dis.

[64] Jensen MP, Karoly P, Braver S (1986). The measurement of clinical pain intensity: a comparison of six methods. Pain.

[65] Ferraz MB, Quaresma MR, Aquino LR, Atra E, Tugwell P, Goldsmith CH (1990). Reliability of pain scales in the assessment of literate and illiterate patients with rheumatoid arthritis. J Rheumatol.

[66] Scott J, Huskisson EC (1979). Vertical or horizontal visual analogue scales. Ann Rheum Dis.

[67] Farrar JT, Young JP, LaMoreaux L, Werth JL, Poole RM (2001). Clinical importance of changes in chronic pain intensity measured on an 11-point numerical pain rating scale. Pain.

[68] Jensen MP, Turner JA, Romano JM, Fisher LD (1999). Comparative reliability and validity of chronic pain intensity measures. Pain.

[69] Eberhart LH, Hogel J, Seeling W, Staack AM, Geldner G, Georgieff M (2000). Evaluation of three risk scores to predict postoperative nausea and vomiting. Acta Anaesthesiol Scand.

[70] Gan TJ (2006). Risk factors for postoperative nausea and vomiting. Anesth Analg.

[71] Gan TJ, Meyer TA, Apfel CC (2007). Chung F, Davis PJ, Habib AS, Hooper VD, Kovac AL, Kranke P, Myles P, Philip BK, Samsa G, Sessler DI, Temo J, Tramer MR, Vander Kolk C, Watcha M, Society for Ambulatory Anesthesia. Society for Ambulatory Anesthesia guidelines for the management of postoperative nausea and vomiting. Anesth Analg.

[72] Meißner W. QUIPS Ergebnis-Fragebogen. http://www.quips-projekt.de/sites/quips/files/QUIPS-Fragebogen%20Ergebnis%20Version%203%203.pdf. Accessed 29 Jan 2019.

[73] Rothaug J, Mescha S, Zwacka S, Göttermann A, Meißner W. Outcome-Parameter in der Akutschmerztherapie - Validierung im Rahmen von Qualitätssicherung in der postoperativen Schmerztherapie. http://www.quips-projekt.de/sites/quips/files/Poster/2006_DAC_BM_Rothaug.pdf. Accessed 29 Jan 2019.

[74] Meissner W, Zwacka S, Rothaug J, Mescha S, Zimmer A, Göttermann A, Uhlig T, Gießer J. QUIPS - Quality improvement in postoperative pain management. http://www.quips-projekt.de/sites/quips/files/Poster/2006_ESA_BM_Poster.pdf. Accessed 29 Jan 2019.

[75] U.S. Department of Health and Human Services FDA Center for Drug Evaluation and Research, U.S. Department of Health and Human Services FDA Center for Biologics Evaluation and Research, U.S. Department of Health and Human Services FDA Center for Devices and Radiological Health (2006). Guidance for industry: patient-reported outcome measures: use in medical product development to support labeling claims: draft guidance. Health Qual Life Outcomes.

[76] Weldring T, Smith SM (2013). Patient-reported outcomes (PROs) and patient-reported outcome measures (PROMs). Health Serv Insights.

[77] Patientenakademie E (2016). Beurteilung der Patient Reported Outcomes (PROs).

[78] Pusic AL, Chen CM, Cano S, Klassen A, McCarthy C, Collins ED, Cordeiro PG (2007). Measuring quality of life in cosmetic and reconstructive breast surgery: a systematic review of patient-reported outcomes instruments. Plast Reconstr Surg.

[79] Messick S (1993). Foundations of validity: meaning and consequences in psychological assessment; ETS research report series.

[80] Deshpande PR, Rajan S, Sudeepthi BL, Abdul Nazir CP (2011). Patient-reported outcomes: a new era in clinical research. Perspect Clin Res.

[81] Beaulieu J, Scutchfield FD, Kelly AV (2003). Content and criterion validity evaluation of National Public Health Performance Standards measurement instruments. Public Health Rep.

[82] Georgalas C, Babar-Craig H, Arora A, Narula A (2007). Health outcome measurements in children with sleep disordered breathing. Clin Otolaryngol.

[83] Apfel CC, Kranke P, Katz MH, Goepfert C, Papenfuss T, Rauch S, Heineck R, Greim CA, Roewer N (2002). Volatile anaesthetics may be the main cause of early but not delayed postoperative vomiting: a randomized controlled trial of factorial design. Br J Anaesth.

[84] Scuderi PE, Conlay LA (2003). Postoperative nausea and vomiting and outcome. Int Anesthesiol Clin.

[85] Marshall PA (1990). Cultural influences on perceived quality of life. Semin Oncol Nurs.

[86] Kagawa-Singer M, Padilla GV, Ashing-Giwa K (2010). Health-related quality of life and culture. Semin Oncol Nurs.

